# Mining TCGA Data Using Boolean Implications

**DOI:** 10.1371/journal.pone.0102119

**Published:** 2014-07-23

**Authors:** Subarna Sinha, Emily K. Tsang, Haoyang Zeng, Michela Meister, David L. Dill

**Affiliations:** 1 Department of Computer Science, Stanford University, Stanford, California, United States of America; 2 Biomedical Informatics Program, Stanford University, Stanford, California, United States of America; 3 Computer Science and Artificial Intelligence Laboratory, Massachusetts Institute of Technology, Cambridge, Massachusetts, United States of America; King Abdullah University of Science and Technology, Saudi Arabia

## Abstract

Boolean implications (if-then rules) provide a conceptually simple, uniform and highly scalable way to find associations between pairs of random variables. In this paper, we propose to use Boolean implications to find relationships between variables of different data types (mutation, copy number alteration, DNA methylation and gene expression) from the glioblastoma (GBM) and ovarian serous cystadenoma (OV) data sets from The Cancer Genome Atlas (TCGA). We find hundreds of thousands of Boolean implications from these data sets. A direct comparison of the relationships found by Boolean implications and those found by commonly used methods for mining associations show that existing methods would miss relationships found by Boolean implications. Furthermore, many relationships exposed by Boolean implications reflect important aspects of cancer biology. Examples of our findings include *cis* relationships between copy number alteration, DNA methylation and expression of genes, a new hierarchy of mutations and recurrent copy number alterations, loss-of-heterozygosity of well-known tumor suppressors, and the hypermethylation phenotype associated with IDH1 mutations in GBM. The Boolean implication results used in the paper can be accessed at http://crookneck.stanford.edu/microarray/TCGANetworks/.

## Introduction

Large-scale cancer genome projects including The Cancer Genome Atlas (TCGA) (http://cancergenome.nih.gov/) are generating an unprecedented amount of multidimensional data using high-resolution microarray and next-generation sequencing platforms. There are opportunities for mining these data sets that can yield insights that would not be apparent from smaller, less diverse data sets. Obtaining the full value of these data requires the ability to find associations between heterogeneous data types.

**Figure 1:**
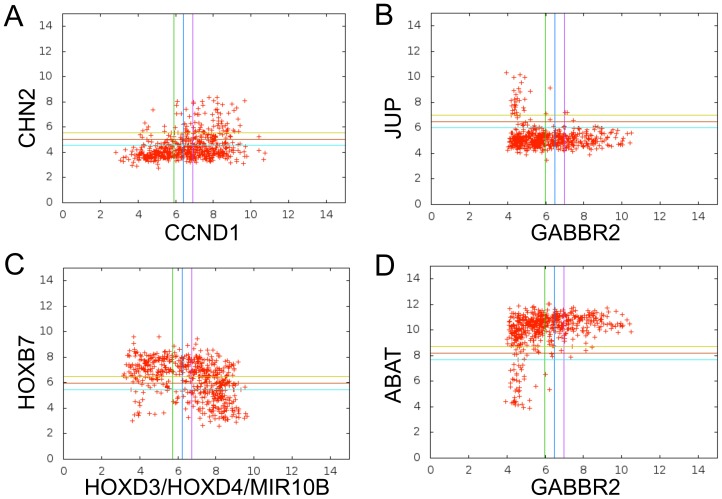
Boolean Implications illustrated using data from gene expression arrays. Each variable has a threshold, represented in the plot as a blue line, that divides the variable into “low” and “high” levels. The green and purple lines are −0.5 and +0.5 away from the threshold on the X axis, respectively. Samples that fell between the green and purple vertical lines on the X axis and between the yellow and blue horizontal lines on the Y axis were not considered during the generation of a Boolean implication. Each point in the scatterplot represents the values of two variables in a tumor sample. Four L-shaped relationships of gene expression are shown (left-to-right and top-to-bottom) (A) LOLO (if CCND1 is low, then CHN2 is low), (B) HILO (if GABBR2 is high, then JUP is low) (C) LOHI (if HOXB7 is low, then HOXD3 is high) (D) HIHI (if GABBR2 is high, then ABAT is high). A Boolean implication exists between two variables when one quadrant is very sparse. Boolean implications can capture L-shaped relationships as well as linear relationships (in which case the two opposite quadrants are sparse), revealing many associations not found by other methods.

In this paper, we propose to use Boolean implications [1] to find pairwise associations in heterogeneous cancer data sets. Boolean implications are if-then rules. The distribution of points in a scatterplot of two variables in a Boolean implication is L-shaped instead of linear (Figure 1). There are four Boolean implications: (1) A-low 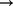 B-low (LOLO), (2) A-high 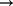 B-low (HILO), (3) A-low 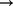 B-high (LOHI), (4) A-high 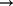 B-high (HIHI). Boolean implications can also be interpreted according to set theory. The Boolean implication A-high 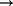 B-high means that “the set of samples where A is high is a subset of the set of samples where B is high”. The implication A-high 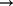 B-low means that “the set of samples where A is high is mutually exclusive with the set of samples where B is high”. Thus far, Boolean implications have been mainly used to analyze gene expression data with a focus on understanding development [2], [3]. Previous work [1] showed that a large number of Boolean implications are present in gene expression data. Based on this finding, we hypothesized that Boolean implications would be useful in the context of mining heterogeneous cancer data sets because (1) they can expose subset and mutual exclusion relationships, both of which have L-shaped scatterplots between related variable pairs; and (2) they provide a common and unified framework to expose relationships between categorical and continuous data. Accordingly, we adapted the existing Boolean implications framework to enable extraction of Boolean implications between mutation, copy number alteration, DNA methylation and gene expression for two large TCGA data sets. Our experiments show that large numbers of implications exist. Experimental comparisons with existing methods to identify pairwise associations in biological data [4]–[10] such as *t* test, correlation and Fisher's exact test revealed that many Boolean implications are missed by other methods. Furthermore, many of the relationships found by Boolean implications captured key aspects of cancer biology.

## Materials and Methods

### Data sets

Our proposed method was applied to two TCGA data sets: glioblastoma (GBM) and ovarian serous cystadenoma (OV). For both, we used the mutation, copy number, DNA methylation and expression data. We started our analysis with the Level 3 data downloaded from the TCGA website (https://tcga-data.nci.nih.gov/tcga/dataAccessMatrix.htm). The GBM data set included 126 patients with mutation and copy number data, 235 patients with methylation and expression data, and 86 patients with mutation, copy number and expression data. The OV data set had 314 patients with mutation and copy number data, and 286 patients with mutation, copy number, methylation and expression data. For expression data for both GBM and OV, we used the U133A array which has 22,277 probes. For methylation data for GBM, we used the Illumina Golden Gate assay which has 1536 probes, and the HumanMethylation27 array which has 27,758 probes. We used the Golden Gate assay only when there were not enough samples with HumanMethylation27 data. For methylation data for OV, we used the HumanMethylation27 array which has 27,758 probes. In GBM, there were 673 mutations and 89,271 copy number alterations. In OV, there were 18,949 mutations and 13,859 copy number alterations.

### Boolean Implication Extraction Between Pairs of Variables

Boolean implications between pairs of variables were detected using a statistical test consisting of two parts: (1) the chi-squared test for independence was used to detect nonrandom associations, (2) then the sparsity test checked for sparseness of a specific quadrant using a maximum-likelihood estimate of the error rate for the points in the sparse quadrant [1]. An implication was considered significant if the first statistic was greater than a cutoff threshold (typically, between 2.0 and 3.0) and the error rate was less than 0.1. The cutoff was chosen to obtain an acceptable false discovery rate (FDR). The details of the FDR test are in the next section. Note that the sparsity test (step 2) distinguishes a Boolean implication from simple non-independence of variables.

### Boolean Implication Generation Integrating Heterogeneous Data

Each type of data was scaled in the range of 0-16, then a threshold separating high and low values was found. The conversion of the TCGA Level 3 data into high and low values was specific to each data type. The conversion details are summarized in Table 1. More details on the conversion procedure are in the Supporting Information (Text_S1).

**Table 1:** Conversion of Different Data Types to High and Low States.

Data Type	Attribute	Transformation	High State in Sample	Low State in Sample
Gene expression	Gene expression probe set	RMA [35] Normalization	Value 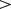 StepMiner [34] threshold	Value < StepMiner threshold
DNA methylation	CpG site	Scaling by a factor of 10	Value 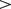 StepMiner threshold	Value < StepMiner threshold
Mutation	Type of mutation per gene	-	Particular type of mutation present in gene	Particular type of mutation absent in gene
Mutation	Mutation per gene	-	Mutation present in gene	Mutation absent in gene
Copy Number	Copy Number amplification per gene	-	Somatic gene amplification present	Somatic gene amplification absent
Copy Number	Copy Number deletion per gene	-	Somatic gene deletion present	Somatic gene deletion absent
Copy Number	Broad Copy Number amplification per segment	-	Broad region amplified	Broad region not amplified
Copy Number	Broad Copy Number deletion per segment	-	Broad Region deleted	Broad Region not deleted

StepMiner [34], which fits patterns of one-step transitions by evaluating every possible placement of the transition (or step) and choosing the one that gives the best fit, was used to derive thresholds that divide the data into low and high states.

After the conversion of all categorical and continuous data into Boolean variables, implications were derived between all pairs of variables. Given the large number of attributes and even larger number of potential relationships, it was necessary to evaluate the significance of the relationships discovered by the above algorithm. We used the FDR computation proposed in previous work [1]. The FDR was obtained by randomly permuting the values for each attribute independently, and then extracting the Boolean implications as above. This analysis was repeated 50 times to compute the average number of Boolean implications in the randomized data. The FDR was the ratio of the average number of Boolean implications in the randomized data and the original data. The cutoff thresholds for the Boolean implication test were set to obtain an acceptable FDR.

#### Enhancements to Boolean Implication Extraction

To enable better detection of Boolean implications with the genomic alterations - mutation and broad Copy Number Alteration (CNA) - data, we introduced several improvements to the implication generation process described earlier [1]. The chi-squared test for independence is not appropriate when the expected number of any entry in the contingency table is less than 5. Instead, we used Fisher's exact test to detect nonrandom associations between variables when processing low frequency events such as mutations and copy number alterations. The Boolean implications between genomic alterations were derived by using Fisher's exact test followed by the sparsity test. We also augmented the implication extraction procedure for genomic alterations by adding artificial normal samples. In both data sets, there exist a few genomic alterations that were present in almost all tumor samples (such as 10q23_31 deletions in GBM and TP53 mutations in OV). In order to find implications involving these genomic alterations, we added artificial normal samples (which did not harbor the mutations or copy number alterations) when deriving the implications between genomic alterations. This was an acceptable procedure since, unlike DNA methylation or gene expression, the mutations and chromosomal alterations were very likely to be cancer-specific as germline mutations and CNVs (copy number variations) had been removed by TCGA. The number of tumor samples with mutation and broad CNA data was 124 and 314 for GBM and OV, respectively. Before we derived the implications, we added 10 and 30 artificial normal samples to the GBM and OV data sets, respectively. An implication was considered significant if the 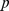-value for the Fisher's exact test was less than a cutoff threshold (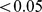) and the error rate was less than 0.14.

## Results and Discussion

In order to mine relationships between variables of different data types such as mutation, copy number, DNA methylation and gene expression, we extracted all four types of Boolean implications (Figure 1). Each attribute was converted to a Boolean variable. The conversion of the TCGA data into high and low values was specific to each data type. The details are summarized in Table 1 (see Materials and Methods for more details). Subsequently, Boolean implications were derived across all pairs of variables. In order to handle low frequency events such as mutations, we made enhancements to the existing Boolean implication extractor [1]. An evaluation of how well the test for Boolean implications performed when picking a known set of implications is demonstrated using a synthetic data set (Text_S2).

The rest of this section describes the Boolean implications we found in the TCGA glioblastoma (GBM) and ovarian serous cystadenoma (OV) data sets and their potential biological significance.

### Numerous Boolean Implications Exist Between Variables of Different Data Types in Glioblastoma and Ovarian Cancer

Table 2 summarizes the number and types of Boolean implications found between variables of different data types in the TCGA GBM and OV data sets. The pairs of data types - {copy number alteration, expression}, {DNA methylation, expression}, {mutation, broad CNAs} and {mutation, DNA methylation} - were mined separately for Boolean implications. Analyzing pairs of data types separately had the advantage of maximizing the number of samples that had information for the data types of interest since not all samples had data for all four types. We only report the number of Boolean implications between variables of different data types as it directly demonstrates the value of integrating different types of data for the same patient. The only exception to this is for the {mutation,broad CNAs} analysis where we report all pairs except the relationships between intra-chromosomal broad CNAs. No additional restrictions, such as the requirement that the variables belong to the same gene, were imposed on the relationships being mined. The FDR for each analysis was 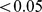, except the {mutation,broad CNAs} analysis in GBM where the FDR was 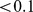 to allow finding interesting relationships with a small sample size. The results clearly indicate that a large number of Boolean implications exist in the data. Furthermore, the implications can be generated in a matter of minutes, demonstrating the scalability of the extraction procedure.

**Table 2:** Summary Table.

Data Set	Data Types Analyzed	HIHI	HILO	LOLO	LOHI
GBM	{Copy Number Alteration, Expression}	202385	178195	52592	15777
	{DNA methylation, Expression}	2875172	1008334	2671091	266135
	{Mutation, Broad CNAs}	170	54	0	0
	{Mutation, DNA methylation}	1057	238	8	0
OV	{Copy Number Alteration, Expression}	271472	823660	8544	1471
	{DNA methylation, Expression}	70498	116200	93543	35934
	{Mutation, Broad CNAs}	75	5	0	0
	{Mutation, DNA methylation}	319	670	33	60

Number of different Boolean implications between variables of different data types in the TCGA GBM and OV data sets. The different data types considered were mutation, copy number, DNA methylation and gene expression. Since chromosomes tended to be altered in large blocks, the copy number data were also organized by chromosomal segments. GISTIC2.0 [36] identifies focal peaks of amplification or deletion as well as broad regions of alteration. The segments derived by GISTIC2.0 are referred to as broad CNAs.

Our results show that a large number of subset (HIHI) and mutual exclusion (HILO) relationships exist between different data types in these large cancer data sets. The next question was to see whether or not these relationships can be found by other methods used for identifying pairwise relationships.

### Other Methods Miss Many Boolean Implications

We compared the relationships identified by Boolean implications to the relationships found by three commonly used bioinformatics techniques for identifying pairwise associations [4]–[9]: *t* test (which looks for a difference of means between two groups), correlation (which looks for linear dependence between two variables) and Fisher's exact test (which looks for non-independence of two variables). For a given set of probes, we extracted all the Boolean implications of a certain kind, say HILO and HIHI, and compared them to the top 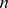 relationships of the other method, where 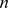 is the number of relationships found by Boolean implications.

We identified relationships using the *t* test and compared them to relationships found by Boolean implications. The *t* test is often used to find the difference in means of a continuous variable between two groups (which is a binary variable). In order for a binary variable 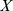 and a continuous variable 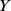 to be related using the *t* test, 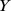 has to be differentially expressed in the samples that have high values of 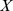 compared with the samples that have low values of 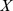. The HILO implications were compared to all relationships found by the *t* test where the continuous variable was under-expressed when the binary variable was high. Similarly, the HIHI implications were compared to all relationships found by the *t* test where the continuous variable was over-expressed when the binary variable was high. The relationships were ranked by 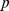-value and the top 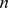 relationships were picked in each case. To make the task more computationally tractable, we did this comparison on 200
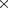
200 pairs chosen at random and repeated the random trials 10 times. Comparisons between the relationships found by the *t* test and the relationships found by Boolean implications showed very little overlap (Table 3). Visual examination of the Boolean implications missing in the top 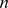 list of the *t* test showed that these relationships were strong L-shaped relationships (see Figures S1–S2 for scatterplots of 20 missed Boolean implications), which represent subset and mutual exclusion relationships. Since the *t* test looks for a difference in means between two groups, it picked out many other relationships that were not necessarily L-shaped (examples in Figures S3–S4).

**Table 3:** Boolean implications versus *t* test.

Type of Implication	Trial No.	Total	Overlap
HILO	1	319	87
	2	216	54
	3	236	72
	4	333	113
	5	329	97
	6	246	80
	7	307	101
	8	233	76
	9	308	63
	10	33	71
HIHI	1	86	21
	2	75	22
	3	126	65
	4	77	27
	5	102	34
	6	73	26
	7	95	33
	8	99	36
	9	81	17
	10	77	28

Comparison between relationships found by Boolean implications and the relationships found by *t* test between randomly selected sets of variables of size 200 in the TCGA OV data set.

To compare Boolean implications with correlation, we computed Pearson's correlation coefficient between the two variables. When comparing with HILO implications, variable pairs with negative Pearson's correlation coefficient were considered. In the comparison with HIHI implications, variable pairs with positive Pearson's correlation coefficients were considered. The pairs were ranked by absolute magnitude of the correlation coefficient in each case, and the top 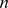 relationships were picked. As in the *t* test case, the comparisons were done on randomly chosen 200
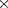
200 variable pairs and the trials were repeated 10 times. The overlap between the relationships ranked highly by correlation and the relationships found by Boolean implications was small (Table 4). Inspecting the Boolean implications that were not in the top 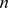 correlation list show that these relationships were strong L-shaped relationships (examples in Figures S5–S6), whereas the relationships picked only by correlation were not (examples in Figures S7–S8). While the comparisons were done using Pearson's correlation coefficient, the results can be generalized to other types of correlation coefficients as well as mutual information since these methods are not tuned to pick out L-shaped relationships.

**Table 4:** Boolean implications versus correlation.

Type of Implication	Trial No.	Total	Overlap
HILO	1	67	9
	2	38	4
	3	83	19
	4	51	2
	5	87	26
	6	80	19
	7	91	15
	8	65	14
	9	64	13
	10	55	5
HIHI	1	44	11
	2	50	4
	3	46	18
	4	48	11
	5	32	2
	6	20	3
	7	47	11
	8	37	7
	9	51	11
	10	61	12

Comparison between relationships found by Boolean implications and the relationships found by correlation between randomly selected sets of variables of size 200 in the TCGA OV data set.

We also compared Boolean implications to the relationships found by Fisher's exact test. For comparison with HILO and HIHI implications, we used the one-tailed Fisher's exact test to compute the statistical significance of mutual exclusion (left tail) or co-occurrence (right tail), respectively. Since Fisher's exact test requires two discrete variables, discretization for continuous variables was done in exactly the same way as for Boolean implications. The top 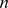 relationships were picked in each case after ranking by 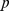-value. As in previous cases, the comparisons were done on randomly chosen 200
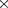
200 variable pairs and the trails were repeated 10 times. Even though the Boolean implication extraction test is most similar to this test, there were differences in the top 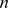 relationships (Table 5). Visual inspection of the top 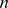 relationships of Fisher's exact test that were not Boolean implications showed that these cannot be reasonably interpreted as subset or mutually exclusive relationships (examples in Figures S9–S10), demonstrating the importance of the sparsity test in extracting Boolean implications.

**Table 5:** Boolean implications versus Fisher's exact test.

Type of Implication	Trial No.	Total	Overlap
HILO	1	239	84
	2	247	84
	3	271	89
	4	245	94
	5	263	91
	6	283	106
	7	305	144
	8	302	125
	9	308	133
	10	273	198
HIHI	1	102	33
	2	86	36
	3	87	14
	4	143	44
	5	70	13
	6	66	11
	7	91	19
	8	76	18
	9	105	29
	10	87	17

Comparison between relationships found by Boolean implications and the relationships found by Fisher's exact test between randomly selected sets of variables of size 200 in the TCGA OV data set.

**Figure 2:**
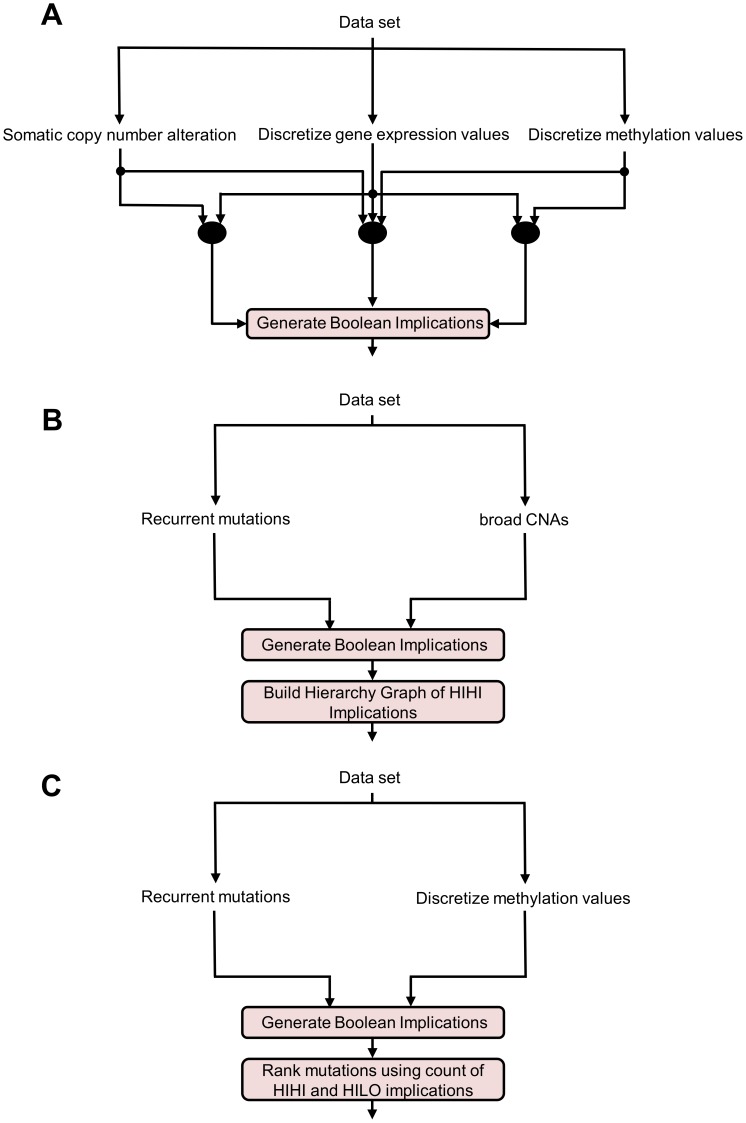
Analysis Pipeline. (A) Boolean implications are extracted between copy number alteration and expression of a gene, DNA methylation and expression of a gene, and Boolean implications combining all three variables: if (gene is deleted or gene is methylated), then gene expression is low. (B) Boolean implications extracted between mutations and recurrent copy number alterations represented as broad Copy Number Alterations (CNAs) are used to build a hierarchy graph. (C) Boolean implications extracted between mutations and methylation are used to predict the role of a mutation in producing aberrant methylation.

The low overlap between the relationships found by Boolean implications and the relationships found by other methods clearly indicate that the relationships found by Boolean implications are unique and would be missed by other commonly used methods. The next question was to see whether or not these relationships were biologically meaningful, which we address in subsequent sections. Figure 2 provides a pictorial summary of the different implications we analyzed to answer the question of biological relevance.

### Boolean Implications Expose *cis*-Regulatory Mechanisms in Glioblastoma and Ovarian Cancer

A common concern in cancer research is to understand the relationship between expression of a gene and somatic copy number alteration and/or DNA methylation. Boolean implications can be used to explore such *cis*-regulatory relationships. Thus, we focused on implications between copy number alteration and/or DNA methylation of a gene and expression of the same gene.

**Figure 3:**
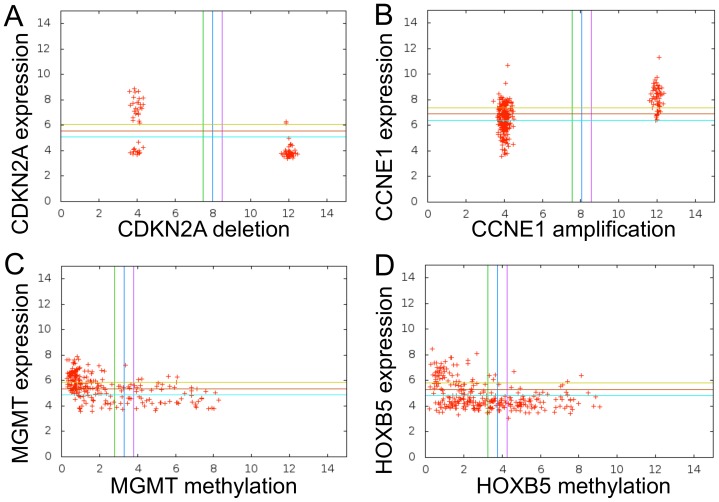
Boolean Implications Between Copy Number Alterations/DNA Methylation and Expression of the Same Gene. Boolean implications between variables are easily verified by inspecting scatter plots. Data for deletions and amplifications were rescaled: a value of 12 implies gene deletion or amplification; a value of 4 implies no somatic copy number change for the gene. Gaussian noise was added so the points do not fall exactly on 4 and 12 to allow easier visualization. The beta-values of methylation (which is how TCGA reports methylation data) were scaled by a factor of 10. (A) HILO Boolean Implication between CDKN2A deletion and CDKN2A expression in TCGA GBM data set. (B) HIHI Boolean Implication between CCNE1 amplification and CCNE1 expression in TCGA OV data set. (B) HILO Boolean Implication between MGMT methylation and MGMT expression in TCGA GBM data set. (D) HILO Boolean Implication between HOXB5 methylation and HOXB5 expression in TCGA OV data set.

Figures 3A and 3B illustrate examples of Boolean implications between copy number alteration and expression of the same gene in both data sets. Figure 3A clearly shows that CDKN2A expression was low when CDKN2A was deleted. Similarly, CCNE1 amplification implied high expression (Fig. 3B). Figures 3C and 3D illustrate examples of Boolean implications between DNA methylation and expression. In both cases, the gene's expression was low when methylation was high. In all of these examples, the relationships were L-shaped: the change in copy number or gene methylation caused expression to be either high or low, but normal copy number and absence of methylation did not produce the opposite expression pattern. Therefore, the L-shape indicates that there are alternate mechanisms that induce the same expression profiles as gene methylation, amplification or deletion.

Table 6 summarizes the number of genes in the GBM and OV data sets where copy number alteration or methylation was related to gene expression. The details of the genes found and the relationship types are in the Supporting Information (Tables S1–S4). Genes that were both amplified and deleted in different groups of samples were most likely passenger events and were excluded from the analysis. Breaking down the analysis further, there were 107 Boolean implications involving gene deletions and 12 involving gene amplifications for the GBM data set. For the OV data set, there were 1703 Boolean implications involving gene deletions and 74 Boolean implications involving gene amplifications. Comparative analysis with existing methods to find *cis*-relationships such as *t* test and correlation showed that the genes found by Boolean implications were often not found by other methods (Table 7), much like the results shown when all relationships were considered.

**Table 6:** Summary of *cis*-regulatory analysis using Boolean implications.

Data Set	Data Types Analyzed	Number of Genes with Implications	Disease-Relevant Gene Sets Found
GBM	{Copy Number Alteration, Expression}	119	TCGA_GLIOBLASTOMA_COPY_NUMBER_UP, PARENT_MTOR_SIGNALING_UP, SA_G1_AND_S_PHASES, BIOCARTA_G1_PATHWAY
GBM	{DNA methylation, Expression}	304	VERHAAK_GLIOBLASTOMA_PRONEURAL, NOUSHMEHR_GBM_SILENCED_BY_METHYLATION,
			BENPORATH_ES_WITH_H3K27ME3, PTEN_DN.V2_UP,
			P53_DN.V1_DN
OV	{Copy Number Alteration, Expression}	1777	DNA_REPAIR, LU_EZH2_TARGETS_DN, DANG_BOUND_BY_MYC
OV	{DNA methylation, Expression}	198	HELLER_SILENCED_BY_METHYLATION_UP, SATO_SILENCED_BY_METHYLATION_IN_PANCREATIC_CANCER_1,
			ONDER_CDH1_TARGETS_2_DN, SMID_BREAST_CANCER_BASAL_DN

Number of genes with relevant Boolean implications between copy number alteration, DNA methylation and expression. List of Disease-Relevant Gene Sets found by GSEA of MSigDB gene sets (FDR q-value 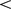
0.05).

**Table 7:** Comparison between genes with *cis*-regulatory relationships found by Boolean implications and alternate methods.

Data Types being Compared	Genes found by Boolean Implications	Alternate Method	Overlap with Alternate Method
{Copy Number Deletion, Expression}	1703	*t* test	860
{Copy Number Amplification, Expression}	74	*t* test	11
{DNA methylation, Expression}	198	correlation	94

Overlap between the genes found by Boolean implications and the top-ranked genes found by an alternate method in the TCGA OV data set.

To better understand these gene lists, we analyzed them with Gene Set Enrichment Analysis (GSEA) [11]. The FDR q-value for finding enriched gene sets was set to 0.05. GSEA analyses of genes found by Boolean implications (Table 6) revealed enrichment for genes that were either downstream of well-known cancer genes or were genes known to play a role in glioblastoma [12], [13] or ovarian cancer [14], [15].

It has been observed in cancer that multiple inactivation events can disrupt the same gene. For instance, the TCGA marker paper on ovarian cancer [7] reported BRCA1 was inactivated by mutually exclusive genomic and epigenomic events. Our framework lends itself to such combinatorial analysis between different data types of the same gene. We describe one such example of combinatorial analysis. Since, both deletions and methylation of genes are considered to have a repressive effect on gene expression, Boolean implications can be used to identify genes that satisfy the following relationship: if (gene is deleted or gene is methylated), then gene expression is low. This query selects genes that are both deleted and methylated (in the same or different samples in the same type of cancer) and that have low expression in either case, potentially indicating that these genes play a central role in the cancer. There were 203 genes and 223 genes that satisfied the stated Boolean implication in the GBM and OV data sets, respectively (Tables S5–S6). This search revealed additional genes where deletion or DNA methylation alone was too rare to yield a statistically significant implication, but the union of the two events produced a significant implication. GSEA analysis of the genes thus identified in the GBM data set overlap with genes that are down-regulated when Wnt signalling is high and when RB1 is down-regulated (WNT_UP.V1_DN, RB_DN.V1_DN). This was in accordance with prior work that showed the importance of the Wnt signalling pathway in gliomas [16] and the significant presence of RB1 mutations in glioblastoma [17]. For the genes found by the query in the OV data set, there was considerable overlap with genes that are down-regulated when CCND1 is over-expressed (CYCLIN_D1_KE_.V1_DN, CYCLIN_D1_UP.V1_DN). CCND1 is known to be over-expressed in epithelial ovarian cancer [18].

To summarize, we found numerous genes with subset and mutual exclusion relationships between copy number alteration, DNA methylation and expression. Combinatorial analysis of data types allowed us to extract additional useful information from the data illustrating an additional benefit of the Boolean implication framework. Furthermore, GSEA analyses revealed that many of these genes were related to glioblastoma and/or ovarian cancer genes. The above analysis shows how one can use Boolean implications to find genes whose expression is regulated by somatic changes in copy number and/or DNA methylation.

### Boolean Implications between Genomic Alterations Reveals Mutual Exclusion, Potential Temporal Progression, Hierarchical Pathway Hits and Loss-of-Heterozygosity (LOH)

The implications between genomic alterations - mutations and broad CNAs - were of two types: HILO and HIHI implications (Table 2). HILO implications represent mutual exclusion. These relationships have been studied previously in the same data sets in the context of pathways [19]. Our analysis found many mutual exclusion relationships between genes on the same pathway and also found new ones since we were not restricted to pathway-specific events (Text_S3).

HIHI implications are approximate subset relations between broad CNAs or between mutations and broad CNAs. While subset relationships are expected when studying regulatory relationships of gene expression, we did not expect to find such subset relations between broad CNAs on different chromosomes or between broad CNAs and gene mutations. Only these unexpected relations were examined further. We chose to investigate these relationships as they have not been studied in the past. The implications between the various genomic alterations may aid in understanding the causes and/or effects of tumorigenesis. Tables containing the complete list of implications are available in the Supporting Information (Tables S7 and S8).

**Figure 4:**
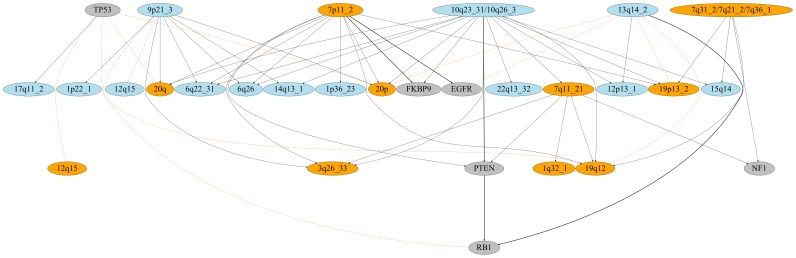
Boolean Implications between Genomic Alterations in GBM. The nodes depicting amplifications, deletions and mutations are colored in orange, blue and grey, respectively. The HIHI implications are represented by black directed edges with the arrow pointing from the superset to the subset. The superset is always above the subset in the diagram. The HILO implications are depicted by red dashed undirected edges. The relationships capture a multitude of biologically interesting phenomena: temporal progression, hierarchical pathway hits, LOH for PTEN and RB1, and a subset relationship between EGFR mutations and 7p11_2 amplifications.

Figure 4, plotted using Graphviz: http://www.graphviz.org, displays a subset of the HIHI Boolean implications between genomic alterations in the GBM data set. The figure was obtained after merging equivalent broad CNAs on the same chromosome. The HILO implications involving broad CNAs that appeared in one or more HIHI implications were overlayed on this graph. The HIHI implications are represented as directed edges from the superset to the subset. The HILO implications are represented as dashed undirected edges. There are several possible reasons for subset-superset relationships: temporal ordering where the superset happens before the subset in cancer progression, hits on the same gene possibly pointing to biologically causal events, pathway related interactions, etc. Examples of all of the above can be found in the HIHI graph. 10q26 deletion is a reported early event in glioamogenesis [20], [21] and mutation of PTEN has been implicated in the malignant progression of astrocytic gliomas [20]. Interestingly, the HIHI graph shows that the 10q23/26 deletions has numerous subsets and is itself not a subset of anything. Furthermore our graph also shows that PTEN mutations have a HIHI implication with deletions of 10q23_31/10q26. This is also consistent with the hypothesis that temporal ordering can give rise to subset relationships. This particular relationship between PTEN mutation and 10q23_31/10q26 deletion also highlights another noteworthy feature of this graph, i.e., implications between mutations and broad CNAs that house the mutated gene. The PTEN locus is 10q23_31. This suggests loss-of-heterozygosity (LOH) of PTEN in the samples that have the PTEN mutation. In fact, the samples with 10q23_31 deletions but no PTEN mutations had several double deletions suggesting that LOH of PTEN is an important event in gliomagenesis. A similar LOH event was observed for RB1: RB1 mutations had a HIHI implication with deletion of 13q14_2, the RB1 locus. An unexpected HIHI implication existed between EGFR mutations and amplification of 7p11_2, the EGFR locus. Thus, samples with EGFR mutated also had EGFR amplified. This suggests strong selective pressure to up-regulate EGFR in some patients with glioblastoma.

Many of the relationships in the graph represent hits on different pathways that need to be dysregulated for cancer progression (Table 8). This analysis used the signalling pathways curated by TCGA [17] (visually depicted in Supplementary Figures 7 and 8 in their paper). We expected to find that most of these relationships would capture hits on two different pathways as is typically expected of co-occuring relationships. However, there are examples of multiple hits on the same pathway indicating complex logic underlying these pathways. Lastly, there were some relationships where two groups of samples that share a genomic alteration had the same pathway hit by different mechanisms. For instance, the 10q23_31 deletion was a superset of 19q12 amplification and RB1 mutation. Both 19q12 amplifications and RB1 mutations affect the RB signalling pathway and did not co-occur in our samples. Hence, the subset relationship between 19q12 amplifications and 10q23_31 deletions and between RB1 mutations and 10q23_31 deletions (via PTEN mutation) could be due to an underlying logical relationship {10q23_31 deletion AND (RB1 mutation OR 19q12 amplification)} governing the development of these genomic alterations in GBM.

**Table 8:** Pathway Analysis of HIHI Boolean Implications.

Subset	Superset	Possible Biological Significance
19q12-amp	7q21_2-amp	CCNE1 and CDK6; double hit on RB signalling pathway
19q12-amp	7q31_2-amp	CCNE1 and MET; hit on RB signalling and RAS pathway
19q12-amp	7p11_2-amp	CCNE1 and EGFR; hit on RB signalling and RAS pathway.
19q12-amp	10q23_31-del	CCNE1 and PTEN; hit on RB signalling and PI3K class 1 pathway
17q11_2-del	TP53_mut	NF1 and TP53; hits on P53 signalling and RAS pathway
PTEN_mut	7p11_2-amp	PTEN and EGFR; hit on P13K class 1 and RAS pathway
PTEN_mut	7q21_2-amp	PTEN and CDK6; hit on PI3K class 1 and RB signalling
RB1_mut	PTEN_mut	RB1 and PTEN; hit on RB and PI3K class 1 pathway
NF1_mut	7q21_2-amp	NF1 and CDK6; hit on RAS and RB signalling
NF1_mut	7q31_2-amp	NF1 and MET; double hit on RAS pathway

Mapping the HIHI implications in the GBM data set to known signalling pathways.

By examining the HIHI implications between the mutations and broad CNAs in ovarian cancer we observed that TP53 mutations and 17q11_2 deletions were primarily supersets in HIHI implications. There is evidence that TP53 mutations, common in ovarian cancer, are an early event in ovarian carcinogenesis [22]. This supports our temporal ordering hypothesis where earlier events are supersets of later events. The 17q11_2 locus houses the NF1 gene, which is known to be important in ovarian cancer. Previous work [23] has found that NF1 defects (due to either mutation or deletion) are associated with activation of the RAS pathway, which is involved in several cancers including ovarian cancer [24]. We also detected a possible LOH event for NF1 since all but one of the samples with NF1 mutations also had 17q11_2 deletions (although it failed our Boolean implication test due to insufficient number of NF1 mutations).

Subset relationships have not been studied in the past and highlight the ability of our method to find novel biologically meaningful relationships.

### Mutation Ranking Reveals Hypermethylator Phenotype Associated with IDH1 Mutations in Glioblastoma

Interesting and somewhat unexpected findings in our initial analysis (Table 2) were the implications between mutations and DNA methylation. Recent work has shown that there exist genetic drivers for DNA hypermethylation in cancer [25]. An interesting question would be to see if genetic drivers of DNA hypermethylation can be identified using the Boolean implications between mutation and DNA methylation. We developed a ranking algorithm by counting the number of HIHI and HILO implications associated with DNA methylation probes with each mutation and ranking the mutations according to the ratio of HIHI to HILO implications. The results of the analysis can be interpreted as follows: if a particular mutation has mainly HIHI implications with methylation probes, then almost all the samples with the mutation have the corresponding probes methylated, suggesting that it plays a role in inducing a hypermethylation phenotype. Similarly, mutations that primarily have HILO implications with methylation probes suggest a role in establishing a hypomethylation phenotype. On the other hand, mutations that have few HIHI or HILO implications with methylation or a combination of HIHI and HILO implications are unlikely to play a dominant role in aberrant DNA methylation.

Interestingly, this analysis placed IDH1 mutation with the largest number of HIHI Boolean implications and also the largest ratio between the number of HIHI and HILO Boolean implication. The mutation has 101 HIHI implications with methylation probes and very few (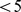) HILO implications with methylation probes. This is in accordance with prior work that IDH1 is a driver of DNA hypermethylation in glioblastoma [26] suggesting that this analysis method can be used to identify novel drivers of aberrant DNA methylation. Applying the same method to the OV data set did not reveal any mutations with aberrant methylation phenotype. It is possible that specific mutations in ovarian serous cystadenoma are not associated with a hypermethylation or hypomethylation phenotype. A quick search of the literature did not reveal any genetic drivers of aberrant DNA methylation in ovarian cancer, confirming our lack of results.

## Summary and Conclusions

We are now moving to an era in which integrating several types of data from high-throughput biological assays will be essential to understand complex diseases such as cancer. Boolean implications provide a conceptually simple and computationally efficient tool for mining subset and mutual exclusion relationships in cancer data. Boolean implications between variables of diverse data types are derived in a matter of minutes. Our results demonstrate that numerous Boolean implications exist between variables of different data types. Furthermore, the relationships found by Boolean implications are not ranked highly by other common bioinformatics methods for identifying pairwise associations. This difference arises from the fact that these methods search for different types of relationships. Thus, Boolean implications provide a complementary approach to already existing methods that identify pairwise associations between random variables.

Since Boolean implications are if-then rules mined from data, there may be a superficial resemblance to association rule mining [27], which finds rules of the form X
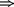
Y, where 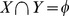. Finding associations (or rules) occurs in two steps: frequent itemset (which refers to a set of items) enumeration and rule generation within each frequent itemset. Since association rule mining has mainly focused on finding higher order relations, the bulk of the research has gone into more efficient algorithms for generating itemsets [28], [29] or a condensed representation of the frequent itemsets [30]. The task of generating itemsets is exponential in the number of variables. Hence, the algorithms cannot scale for data such as the TCGA with hundreds of thousands of variables per sample. So far, application of association rules to biological data has been restricted to simultaneous analysis of gene expression and GO categories [31], [32]. Even if association rules are restricted to itemsets of size two (so they find relationships between pairs of variables), there are issues with the rules generated. The associations generated between pairs of variables are either not statistically significant when they use the support-confidence framework [31] (which has been shown in detail earlier [33]) or they produce mainly co-occurence relationships [32] that are not necessarily logically interpretable as subset or mutual exclusion relationships. Thus, Boolean implications are not comparable to association rules, despite the superficial similarity.

Delving into the biological significance of the extracted Boolean implications demonstrated that subset and mutual exclusion relationships result from a multitude of biological phenomena: *cis*-regulatory mechanisms of gene regulation, temporal ordering, interactions of multiple pathways, loss-of-heterozygosity of tumor suppressors and mutation-specific epigenetic states. Many of these relationships revealed *cis*-regulatory mechanisms where there were subset and mutual exclusion relationships between different data types (copy number alteration, DNA methylation and gene expression) of the same gene (Table 6), either between pairs of variables or in some cases involving variables of all three data types. Subsequent analysis using GSEA demonstrated that the genes obtained by mining these relationships were biologically meaningful and had overlap with several known cancer genes -TP53, PTEN, RB1 in glioblastoma; MYC, EZH2, CCND1, E-cadherin in ovarian cancer, as well as overlap with genes in important signaling pathways - MTOR in glioblastoma and WNT in ovarian cancer. So far, there has been no work on extracting subset relationships between mutations and copy number alterations, and this represents a unique strength of our analysis. The subset relationships between genomic alterations - mutation and broad CNAs- exposed evidence of temporal ordering, loss-of-heterozygosity (LOH) of two well-known tumor suppressors (PTEN and RB1) and interesting pathway related interactions. A well-known relationship between the IDH1 mutation and DNA methylation was found by analyzing the mutation and DNA methylation Boolean implications in GBM, confirming known biology that IDH1 mutation produces hypermethylation in glioblastoma [26]. This finding also demonstrated the value of analyzing a set of related HIHI and HILO Boolean implications. Given the diversity of biological insights that can be derived by looking at a small portion of the entire set of extracted Boolean implications, we speculate that Boolean implications will be an useful tool for mining relationships in cancer data sets with diverse data types and the extracted relationships will lead to novel biological insights.

One concern with using the Boolean abstraction could be the loss of information that discretization can introduce. However, this is not an issue unique to our proposed solution. Many effective statistical techniques discretize data because there are tradeoffs that compensate for the loss of information. In our specific case, the data for mutation is discrete and hence a Boolean abstraction loses very little information. Copy number alterations are inherently discrete but are represented as continuous values in the data. These data are re-discretized to identify copy number gains and losses. The cutoffs for gene expression and methylation are derived in a systematic fashion using StepMiner [34], which looks at the distribution of the data for each expression/methylation probe across all the samples to come up with a threshold. While thresholding the methylation and gene expression data does result in the loss of some information, the development of a common framework to analyze very different data types provides power to the user by allowing combinatorial analysis (copy number, methylation and expression) and revealing unexpected associations (subset relationships between mutation and copy number). There are often concerns raised about the applicability of Boolean abstractions in biology. However, applying Boolean implications to the TCGA glioblastoma and ovarian serous cystadenoma data sets resulted in the re-discovery of some well-known relationships and also found several new relationships between genomic alterations, DNA methylation and gene expression data.

To conclude, Boolean implications are a novel way to explore large data sets and expose numerous subset and mutual relationships, which can lead to new hypotheses for further investigation. The proposed methods are quite general and can be applied to other types of heterogeneous data. We are in the process of applying the above methods to other TCGA data sets. One direction for future work would be to generate higher-order relationships for certain combinations of variables which are biologically meaningful. Other areas of future investigation would be to use the relationships found by Boolean implications along with other data to answer specific biological questions. One such example would be to combine the subset relationships between genomic alterations with additional data to identify novel temporal ordering relationships in cancer progression.

## Supporting Information

Figure S1 **Examples of HILO Boolean implications that were not ranked highly in a** ***t*** **test based approach to find relationships between the same variable pairs.**
(TIF)

Figure S2 **Examples of HIHI Boolean implications that were not ranked highly in a** ***t*** **test based approach to find relationships between the same variable pairs.**
(TIF)

Figure S3 **Examples of non L-shaped relationships found by the** ***t*** **test based approach.** These were prioritized over several L-shaped relationships picked out by HILO Boolean implications.
(TIF)

Figure S4 **Examples of non L-shaped relationships found by the** ***t*** **test based approach.** These were prioritized over several L-shaped relationships picked out by HIHI Boolean implications.
(TIF)

Figure S5 **Examples of HILO Boolean implications that were not ranked highly by the correlation based approach to find relationships between the same variable pairs.**
(TIF)

Figure S6 **Examples of HIHI Boolean implications that were not ranked highly by the correlation based approach to find relationships between the same variable pairs.**
(TIF)

Figure S7 **Non L-shaped relationships found by the correlation based approach.** These were prioritized over several L-shaped relationships picked out by HILO Boolean implications.
(TIF)

Figure S8 **Non L-shaped relationships found by the correlation based approach.** These were prioritized over several L-shaped relationships picked out by HIHI Boolean implications.
(TIF)

Figure S9 **Examples of non L-shaped relationships found by Fisher's exact test.** These were prioritized over several L-shaped relationships picked out by HILO Boolean implications.
(TIF)

Figure S10 **Examples of non L-shaped relationships found by Fisher's exact test.** These were prioritized over several L-shaped relationships picked out by HIHI Boolean implications.
(TIF)

Table S1 **Genes that have Boolean implications between copy number alterations and gene expression in the TCGA GBM data set.**
(TXT)

Table S2 **Genes that have Boolean implications between copy number alterations and gene expression in the TCGA OV data set.**
(TXT)

Table S3 **Genes that have Boolean implications between methylation and gene expression in the TCGA GBM data set.**
(TXT)

Table S4 **Genes that have Boolean implications between methylation and gene expression in the TCGA OV data set.**
(TXT)

Table S5 **Genes that are found by looking at both deletions and methylation in the TCGA GBM data set, excluding those found by methylation.**
(TXT)

Table S6 **Genes that are found by looking at both deletions and methylation in the TCGA OV data set.**
(TXT)

Table S7 **HIHI and HILO implications between mutations and broad CNAs in the TCGA GBM data set.**
(TXT)

Table S8 **HIHI and HILO implications between mutations and broad CNAs in the TCGA OV data set.**
(TXT)

Text S1 **Conversion of Different TCGA Data Types to Boolean Values.**
(PDF)

Text S2 **Boolean Implication Extraction on a Synthetic Data Set.**
(PDF)

Text S3 **Mutual Exclusion Relations in the Genomic Alterations Sub-network.**
(PDF)
