# Supplementary figures and images for: Mining TCGA Data Using Boolean Implications
Source: PLoS One. 2014 Jul 23;9(7):e102119. doi: 10.1371/journal.pone.0102119 (PMC4108374; doi:10.1371/journal.pone.0102119)

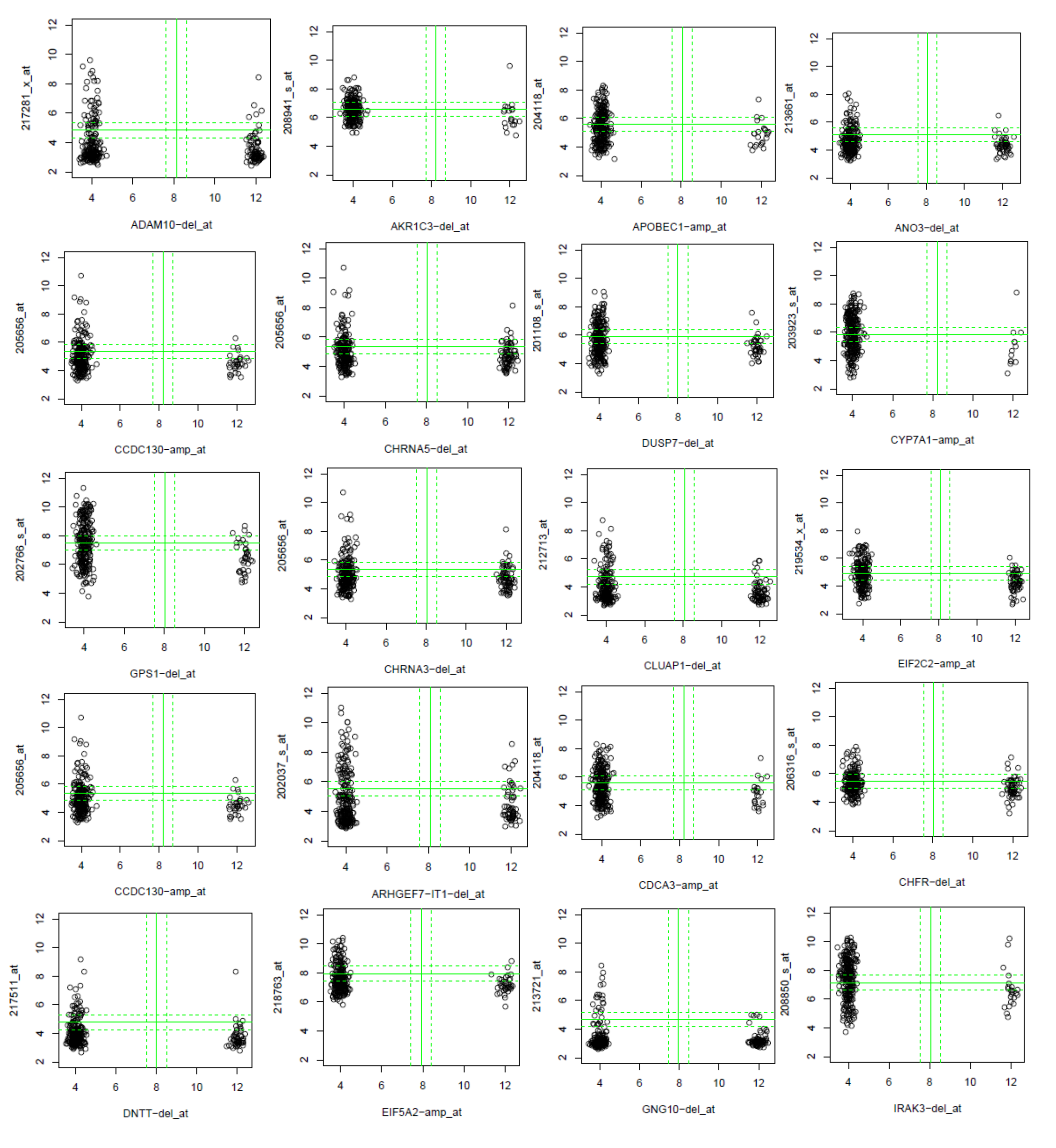

Supplement: Figure S1 — Examples of HILO Boolean implications that were not ranked highly in a t test based approach to find relationships between the same variable pairs. (TIF) [file pone.0102119.s001.tif]

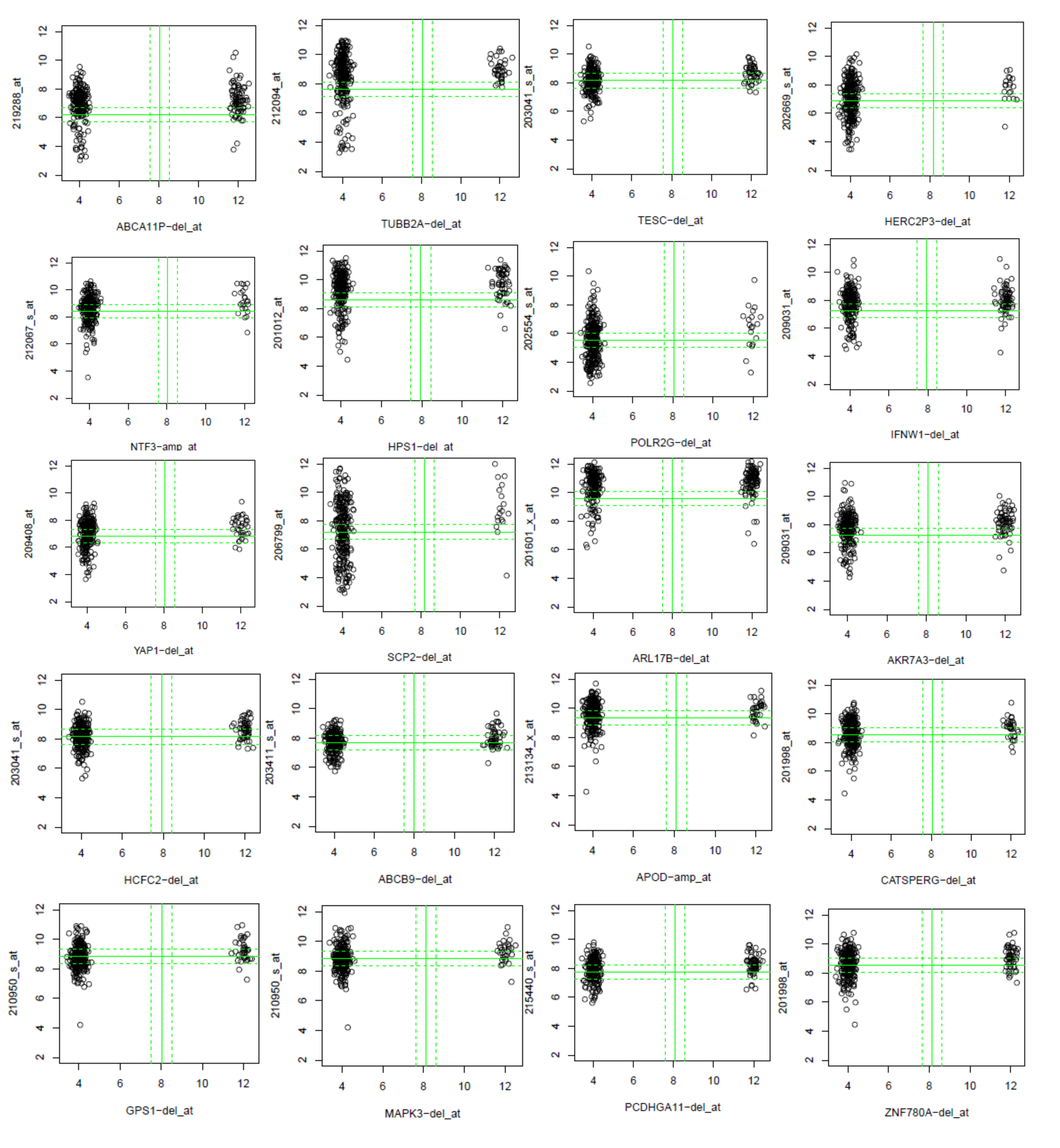

Supplement: Figure S2 — Examples of HIHI Boolean implications that were not ranked highly in a t test based approach to find relationships between the same variable pairs. (TIF) [file pone.0102119.s002.tif]

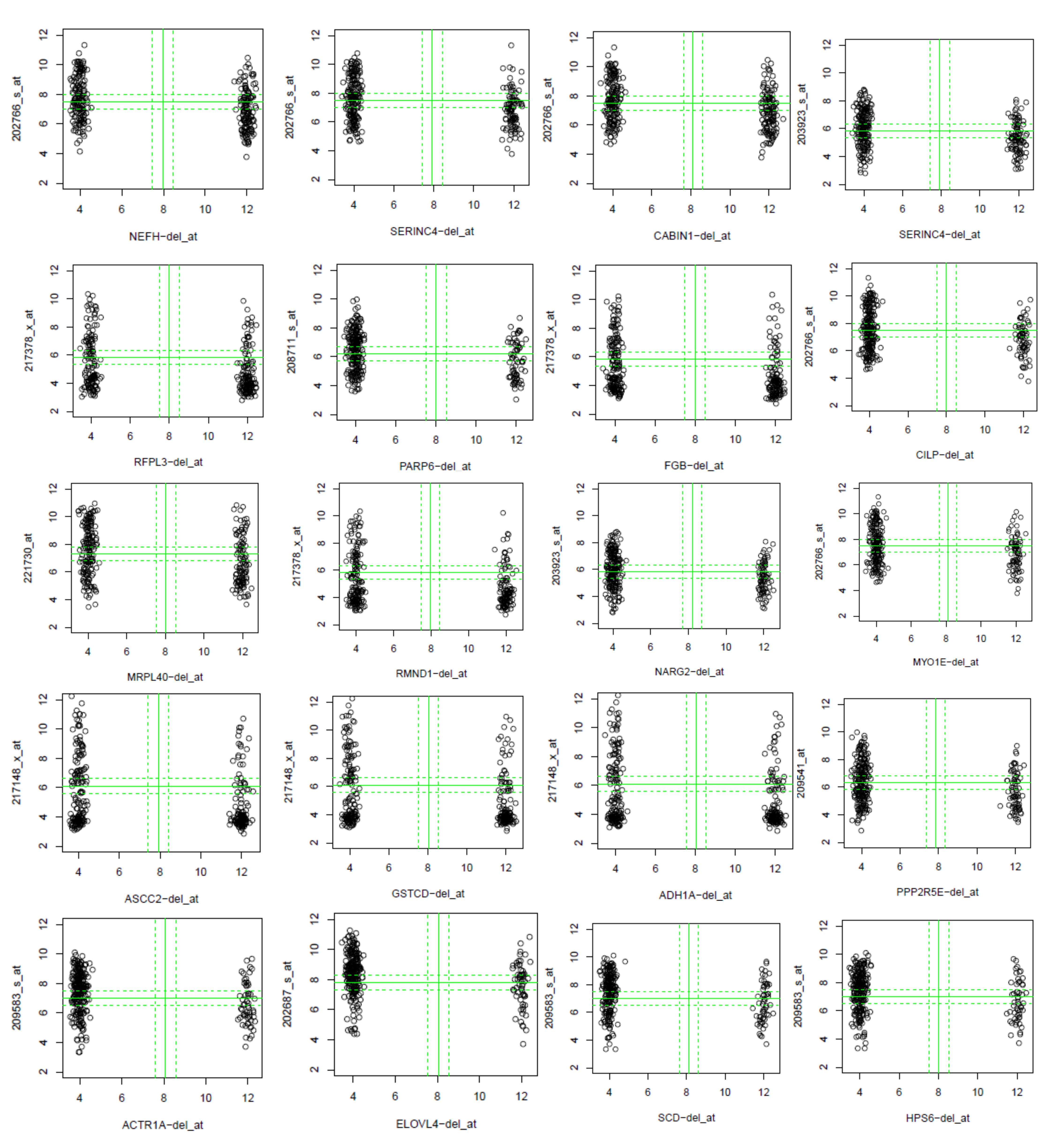

Supplement: Figure S3 — Examples of non L-shaped relationships found by the t test based approach. These were prioritized over several L-shaped relationships picked out by HILO Boolean implications. (TIF) [file pone.0102119.s003.tif]

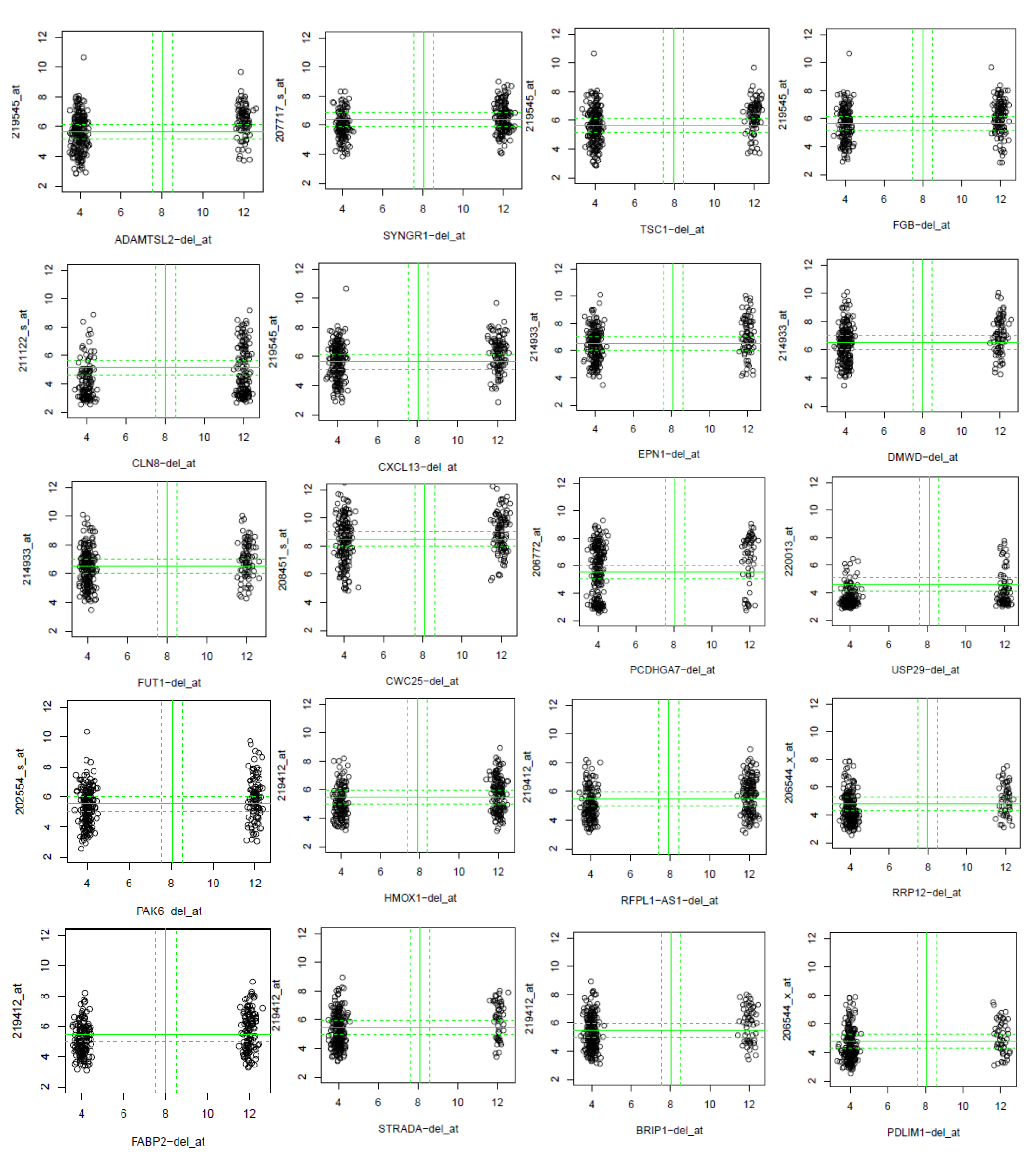

Supplement: Figure S4 — Examples of non L-shaped relationships found by the t test based approach. These were prioritized over several L-shaped relationships picked out by HIHI Boolean implications. (TIF) [file pone.0102119.s004.tif]

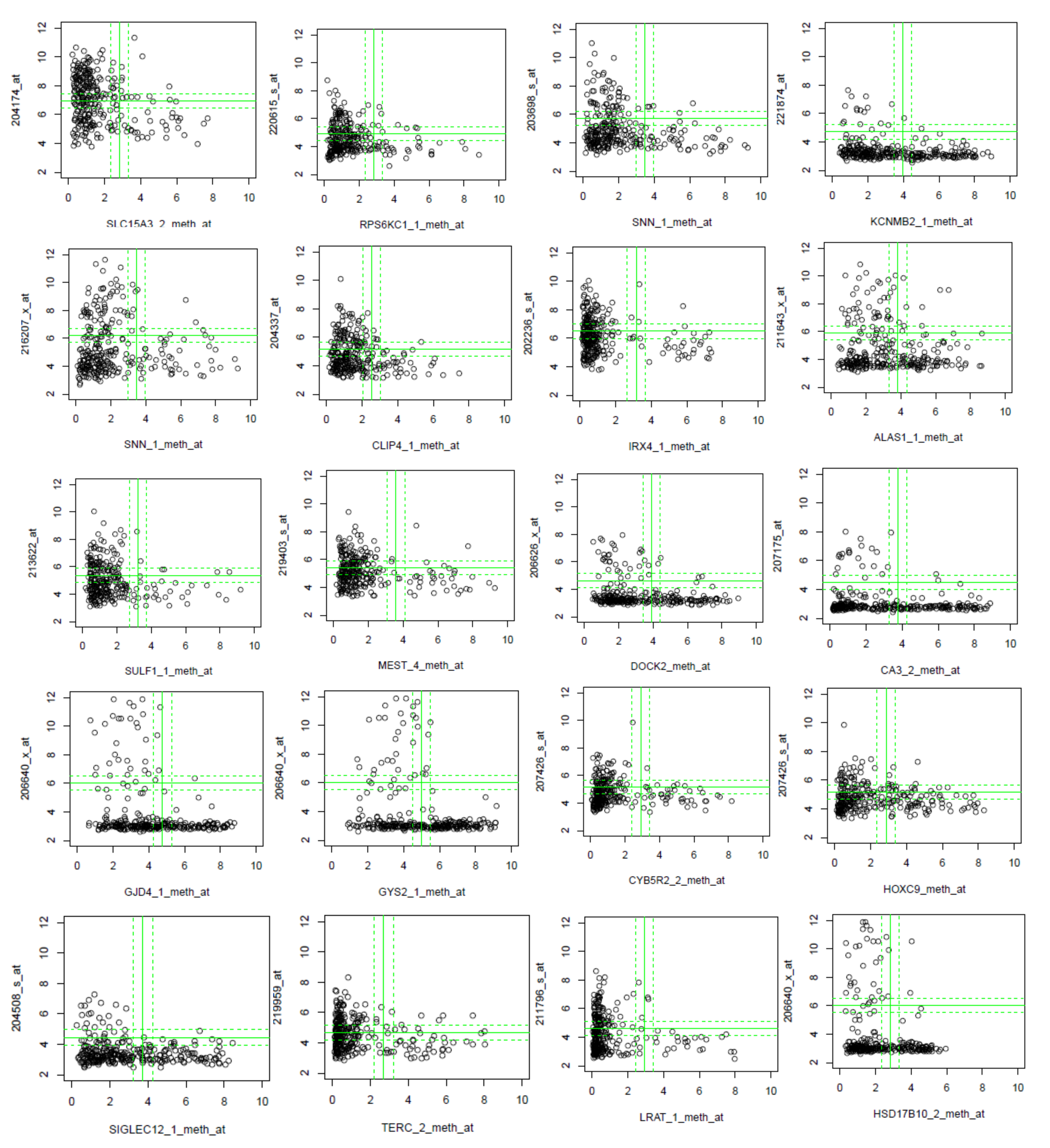

Supplement: Figure S5 — Examples of HILO Boolean implications that were not ranked highly by the correlation based approach to find relationships between the same variable pairs. (TIF) [file pone.0102119.s005.tif]

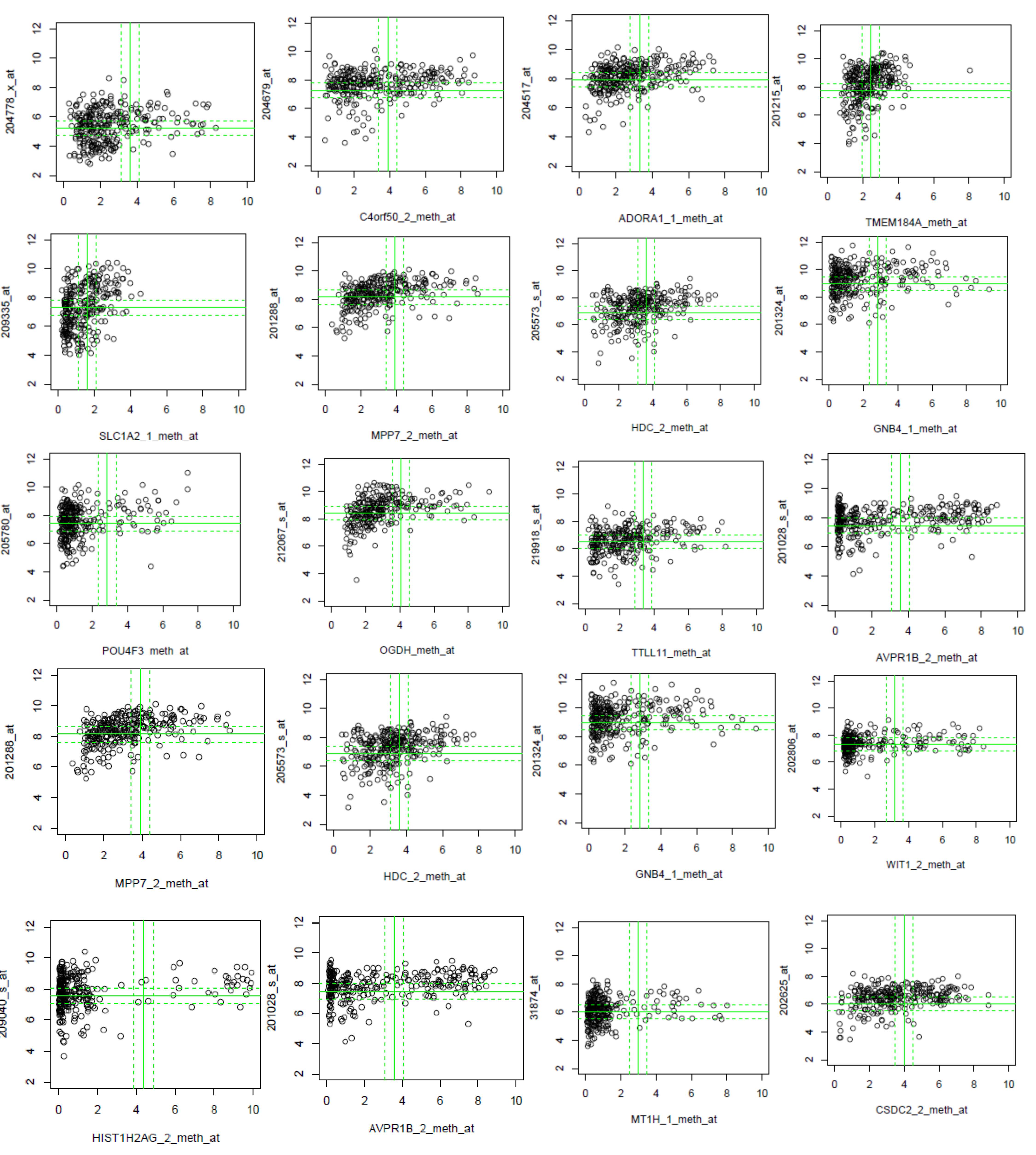

Supplement: Figure S6 — Examples of HIHI Boolean implications that were not ranked highly by the correlation based approach to find relationships between the same variable pairs. (TIF) [file pone.0102119.s006.tif]

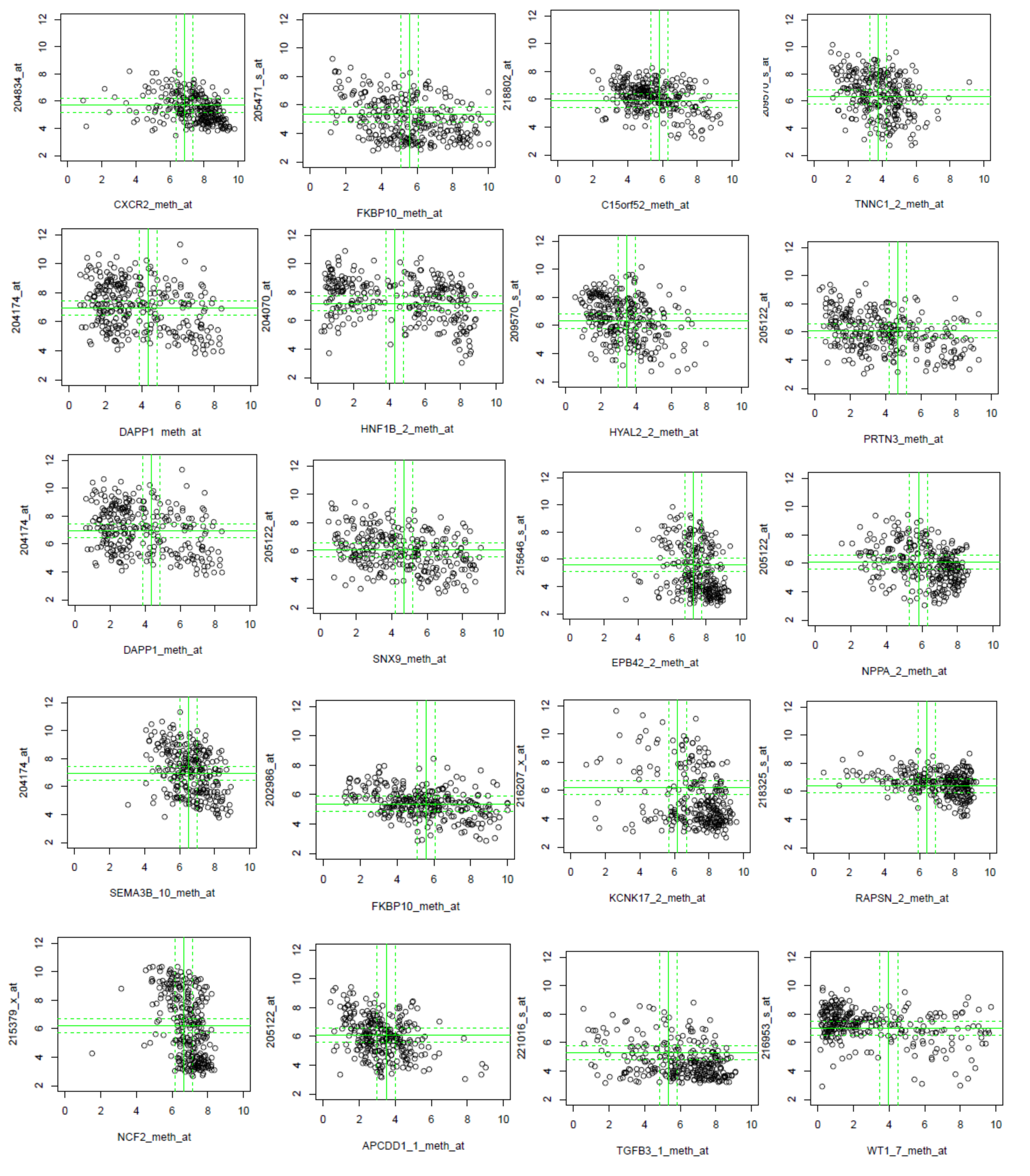

Supplement: Figure S7 — Non L-shaped relationships found by the correlation based approach. These were prioritized over several L-shaped relationships picked out by HILO Boolean implications. (TIF) [file pone.0102119.s007.tif]

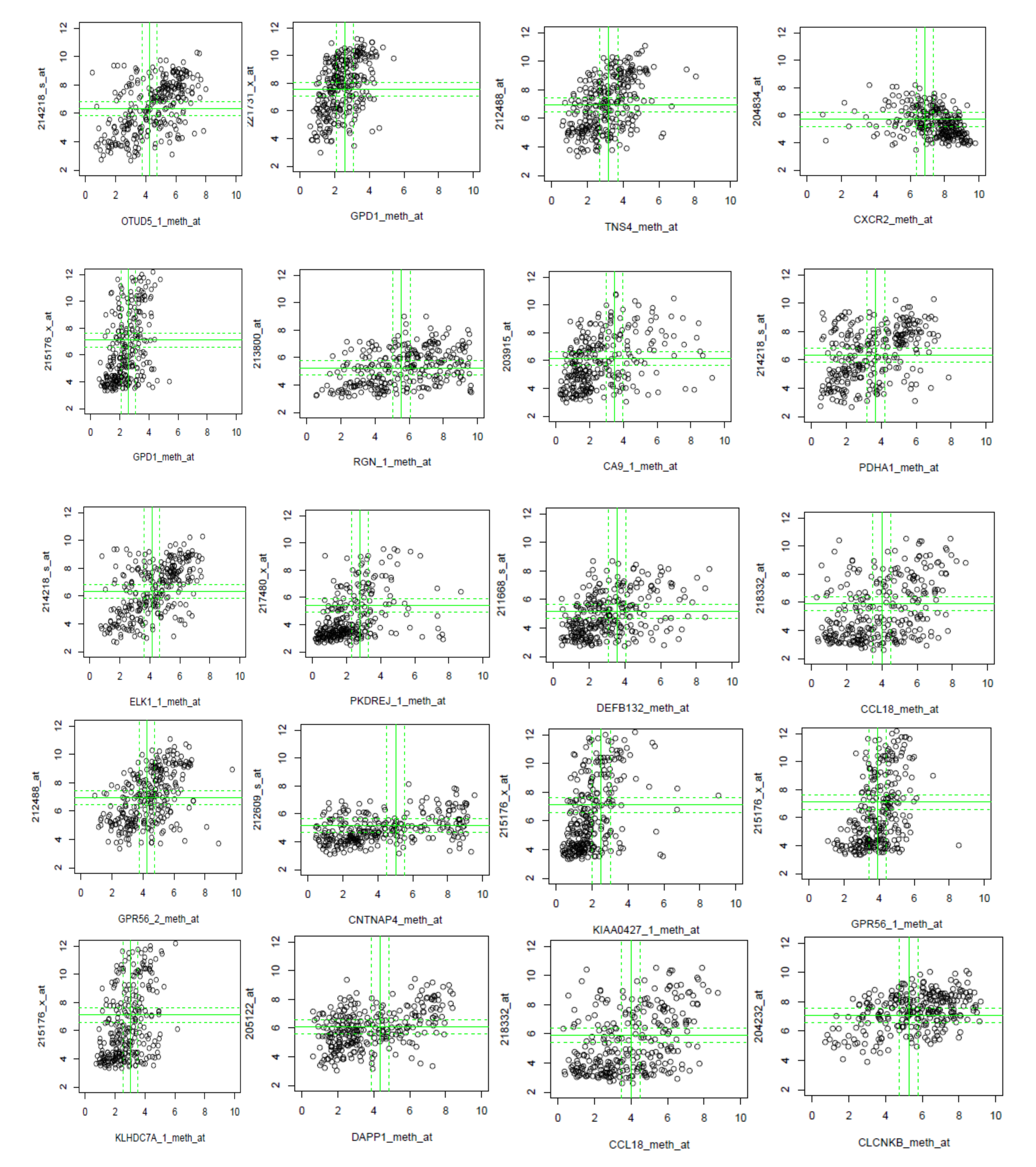

Supplement: Figure S8 — Non L-shaped relationships found by the correlation based approach. These were prioritized over several L-shaped relationships picked out by HIHI Boolean implications. (TIF) [file pone.0102119.s008.tif]

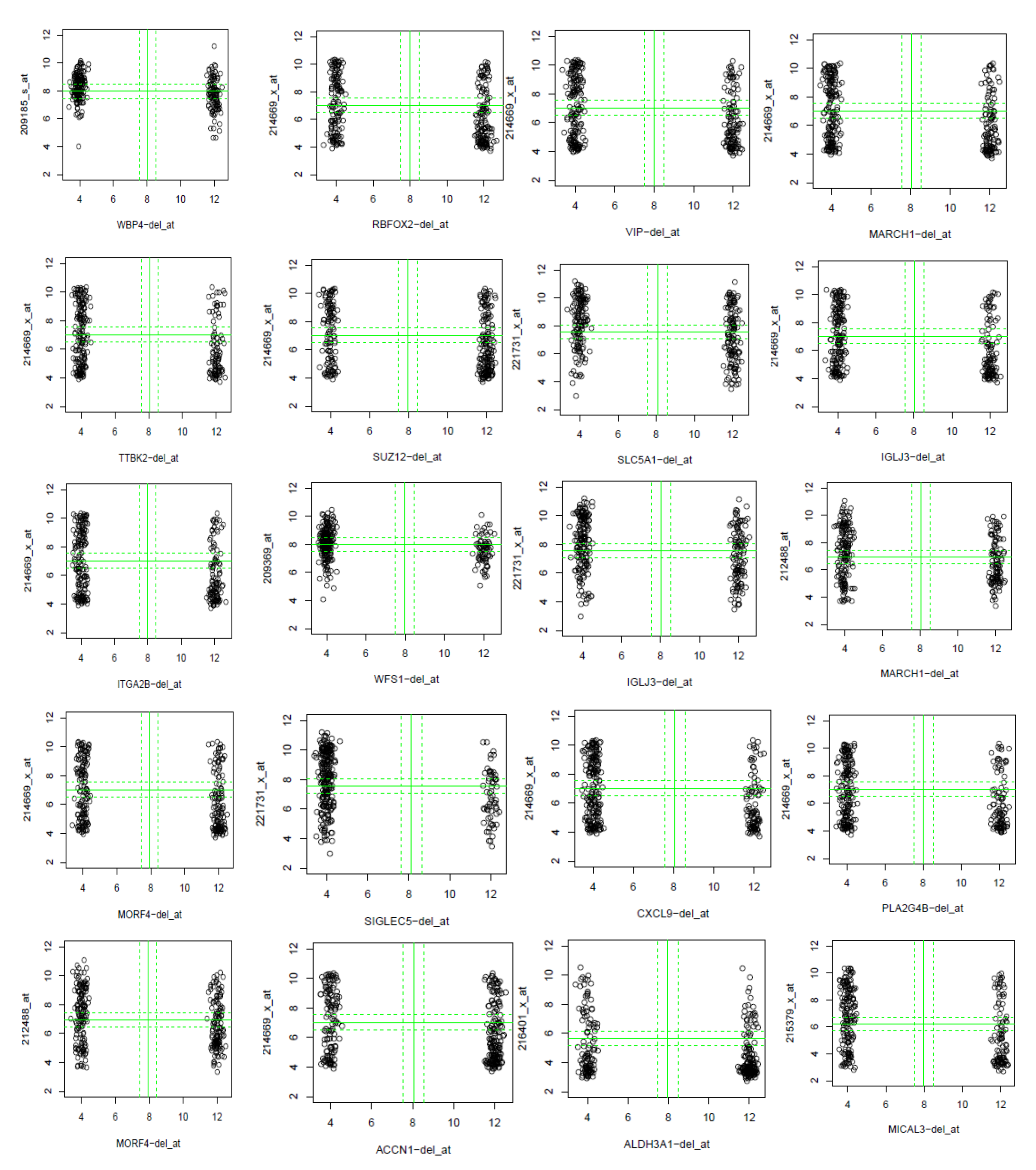

Supplement: Figure S9 — Examples of non L-shaped relationships found by Fisher's exact test. These were prioritized over several L-shaped relationships picked out by HILO Boolean implications. (TIF) [file pone.0102119.s009.tif]

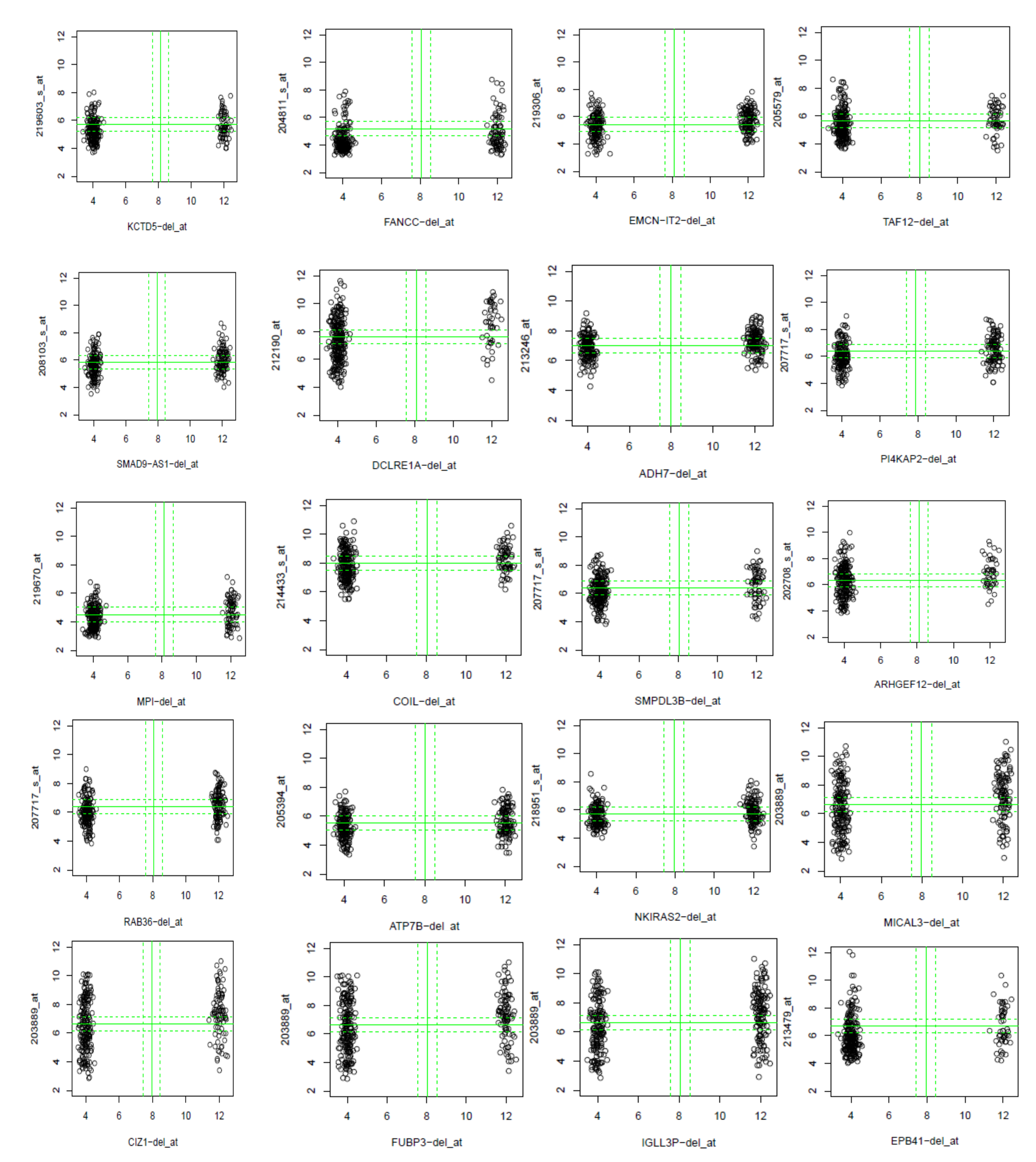

Supplement: Figure S10 — Examples of non L-shaped relationships found by Fisher's exact test. These were prioritized over several L-shaped relationships picked out by HIHI Boolean implications. (TIF) [file pone.0102119.s010.tif]
