# Supplementary material for: Mining TCGA Data Using Boolean Implications
Source: PLoS One. 2014 Jul 23;9(7):e102119. doi: 10.1371/journal.pone.0102119 (PMC4108374; doi:10.1371/journal.pone.0102119)
Supplement: Text S1 — Conversion of Different TCGA Data Types to Boolean Values. (PDF) [file pone.0102119.s019.pdf]

Conversion of Different TCGA Data Types to Boolean Values

The conversion of the Level 3 TCGA data into Boolean variables was specific to each data type.

Mutation. The mutation data specify the mutated genes and the mutation type on a per sample basis. A Boolean variable was introduced for each mutated gene. Boolean variables were also introduced for each type of mutated gene (such as frame shift deletions, missense mutations, nonsense mutations, and splice site mutations). For each sample, the Boolean variable associated with a given mutation was set to high if the mutation associated with the variable was present and to low otherwise.

Copy Number Alteration (Gene Level). The data for copy number alterations contain the segmented copy number data for the tumor and normal samples. For each tumor sample, we determined the tumor-specific alterations by removing regions that had more than 50% overlap with altered regions in the corresponding normal sample. The segmented data were provided in the hg18 human genome assembly. We used the liftover [1] program to convert the tumor-specific regions to hg19. We then used the identified regions in hg19 to find, in each tumor sample, the genes affected by a copy number alteration. Next, two Boolean variables were introduced - one for gene amplification and another for gene deletion. For each sample, the Boolean variables for amplification or deletion were high if the gene was amplified or deleted.

Copy Number Alteration (Region Level). Since chromosomes tended to be altered in large chunks, the copy number data were also organized by chromosomal segments. GISTIC2.0 [2] identified focal peaks of amplification or deletion as well as broad regions of alteration. The GISTIC results provided changes in copy number as the absolute copy number minus two on a per sample basis for both focal and broad regions. Amplification or deletion of each broad CNA (Copy Number Alteration) was treated as an attribute of the samples. Boolean variables were introduced for each broad CNA. As with copy number for genes, we used separate variables for amplification and deletion. The Boolean variables for amplified broad CNAs were set to high or low depending on whether or not the magnitude of the change in copy number ≥ 0.5 for each sample. For broad CNA deletions, we used a threshold of -0.5 for the change in copy number. We used the GISTIC results for the TCGA samples from the Broad Institute Firehose (http://gdac.broadinstitute.org/runs/analyses __2012_10_24/reports/cancer/) analysis run from `2012_10_24` (for GBM) and `2012_08_25` (for OV).

Gene Expression. The conversion of gene expression data to Boolean variables was done in previous work [3]. The same procedure was adopted here. First, the gene expression data were normalized using the standard Robust Multi-chip Analysis algorithm [4]. The normalization was done using the `justRMA` function in bioconductor. Then StepMiner [5], which fits patterns of one-step transitions by evaluating every possible placement of the transition (or step) and chooses the one that gives the best fit, was used to derive thresholds that divided the data into low and high states. A Boolean variable was associated with each gene expression probe and was set to high or low depending on whether it was above or below the threshold.

DNA Methylation. The methylation status of the Illumina Golden Gate assay and the Infinium Human Methylation 27k BeadChip assay can be measured as beta-values, which is how the TCGA reported it. For each specific CpG site, the beta-value was calculated from the intensity of the methylated and unmethylated alleles, as the ratio of the fluorescent signals: $beta{-}value = \frac{Max(Methyl,0)}{(Max(Methyl,0)+Max(Unmethyl,0)+100)}$ The beta-value is a continuous variable between 0 (absent methylation) and 1 (completely methylated) representing the ratio of the methylated allele to the combined locus intensity. It can be interpreted as an approximation of the percentage of methylation for a given CpG site in the sample. After appropriately

scaling the beta-values, StepMiner [5] was used to derive thresholds that divide the data into low and high levels of methylation. For each methylation probe, we set the associated Boolean variable to high if the value of the probe was above the threshold. A gene was said to be methylated in a sample when the associated Boolean variable of any probe belonging to the gene had a high value in the same sample.

References

1. Hinrichs A, Karolchik D, Baertsch R, Barber G, Bejerano G, et al. (2006) The ucsc genome browser database: update 2006. Nucleic Acids Research 1(34): D590-598.

2. Mermel C, Schumacher S, Hill B, Meyerson M, Beroukhim R, et al. (2011) Gistic2.0 facilitates sensitive and confident localization of the targets of focal somatic copy-number alteration in human cancers. Genome Biology 12(4): R41.

3. Sahoo D, Dill D, Gentles A, Tibshirani R, Plevritis S (2008) Boolean implication networks derived from large scale, whole genome microarray data. Genome Biology 9: R157.

4. Irizarry R, Bolstad B, Collin F, Cope L, Hobbs B, et al. (2003) Summaries of affymetrix genechip probe level data. Nucleic Acids Research 31(4): e15.

5. Sahoo D, Dill D, Tibshirani R, Plevritis S (2007) Extracting binary signals from microarray time-course data. Nucleic Acids Research 35(11): 3705-372.
