# Supplementary material for: Mining TCGA Data Using Boolean Implications
Source: PLoS One. 2014 Jul 23;9(7):e102119. doi: 10.1371/journal.pone.0102119 (PMC4108374; doi:10.1371/journal.pone.0102119)
Supplement: Text S2 — Boolean Implication Extraction on a Synthetic Data Set. (PDF) [file pone.0102119.s020.pdf]

Boolean Implication Extraction on a Synthetic Data Set

We used a synthetic data set to demonstrate the efficacy of our method for detecting Boolean implications. To generate a data set with a known list of Boolean implications, we used the Boolean circuit shown in Figure 1. The artificial circuit contained many `HIHI` and `LOLO` implications along with two correlated signals. In the absence of noise and with complete coverage of all input combinations, extracting Boolean implications given the various combinations of the inputs and the internal signals is a straightforward task. However, biological data are inherently noisy. Furthermore, complete coverage is lacking, i.e., data for all the input combinations are not always available. Hence, we evaluated our implication detector under the following experimental setup:

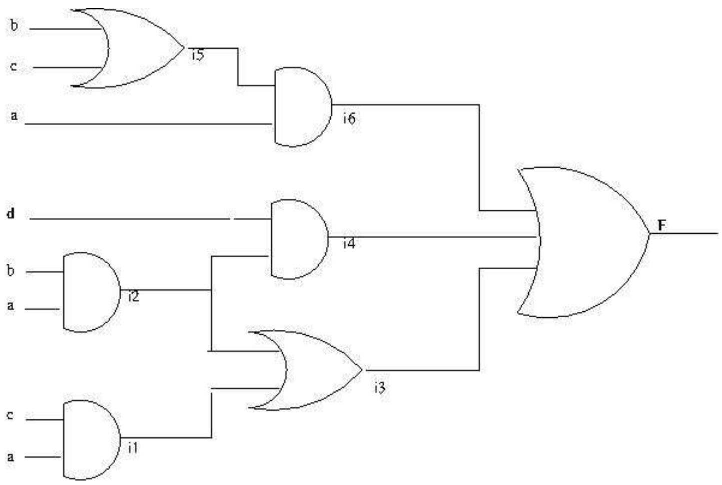

Figure 1. Boolean Circuit used for Implication Generation

1. We simulated the circuit 64 times with input combinations drawn from a uniform distribution.
2. We set the value of a signal to 12 or 4, depending on whether the value was 1 or 0. This gave a 11×64 data frame, where the number of signals was 11 and the number of samples was 64.
3. For each simulation, we added Gaussian noise to all the signals. Three levels of noise were used for the experiments:
   (a) *Error-0*: The mean and standard deviation of the Gaussian noise were 0 and 0.2, respectively.
   (b) *Error-2*: The mean and standard deviation of the Gaussian noise were 0 and 2.0, respectively. In this case, approximately 2% of the values had errors. By errors, we mean that the low values became high values and vice versa compared to the noise-free data frame.
   (c) *Error-10*: The mean and standard deviation of the Gaussian noise were 0 and 4.0, respectively. In this case, approximately 10% of the signals had errors, where an error was as defined in Step 3(b).

Using this synthetic example, we compared the true known set of Boolean implications in the circuit to the Boolean implications extracted by the original method proposed by Sahoo et al. in 2008 (henceforth, referred to as *Original-BR*) and our enhanced method (henceforth, referred to as *New-BR*). For comparison with correlation, we also provide Pearson's correlation coefficient for the signals involved in implication relationships.

Table 1 summarizes the results of our study. Columns 1 and 2 give the pairs of signals that have a true Boolean implication (BI). The type of implication is in Column 3. Columns 4-6 give the results of *Original-BR*, *New-BR* and *Correlation* in the *Error-0* circuit. Similar results for *Error-2* and *Error-10* circuits are captured in Columns 7-9 and Columns 10-12, respectively. It should be noted that even in the absence of any error, a statistical test can fail to pick up an implication if there is lack of enough evidence that an implication exists. The summary of the sensitivity results is given in Table 2. *New-BR* strictly

			Error-0			*Error-2*			*Error-10*		
Signal1	Signal2	BI (True Positives)	BI (*Original-BR*)	BI (*New-BR*)	Correlation	BI (*Original-BR*)	BI (*New-BR*)	Correlation	BI (*Original-BR*)	BI (*New-BR*)	Correlation
a	i1	LOLO	LOLO	LOLO	0.70	LOLO	LOLO	0.54	-	-	0.40
a	i2	LOLO	LOLO	LOLO	0.54	LOLO	LOLO	0.38	LOLO	LOLO	0.51
a	i3	LOLO	LOLO	LOLO	0.81	LOLO	LOLO	0.61	LOLO	LOLO	0.55
a	i4	LOLO	LOLO	LOLO	0.46	LOLO	LOLO	0.33	-	-	0.16
a	i6	LOLO	LOLO	LOLO	0.82	LOLO	LOLO	0.60	LOLO	LOLO	0.55
a	F	LOLO	LOLO	LOLO	0.81	LOLO	LOLO	0.61	-	-	0.48
b	i2	LOLO	LOLO	LOLO	0.47	LOLO	LOLO	0.36	-	-	0.20
b	i4	LOLO	LOLO	LOLO	0.39	LOLO	LOLO	0.38	-	-	0.17
b	i5	HIHI	-	HIHI	0.49	-	HIHI	0.42	-	-	0.33
c	i1	LOLO	LOLO	LOLO	0.49	-	-	0.31	LOLO	LOLO	0.29
c	i5	HIHI	HIHI	HIHI	0.58	HIHI	HIHI	0.39	-	-	0.28
d	i4	LOLO	-	LOLO	0.33	-	LOLO	0.29	-	-	0.23
i1	i3	HIHI	HIHI	HIHI	0.85	HIHI	HIHI	0.66	HIHI	HIHI	0.42
i1	i5	HIHI	-	HIHI	0.30	-	HIHI	0.24	-	HIHI	0.16
i1	i6	HIHI	HIHI	HIHI	0.86	HIHI	HIHI	0.57	HIHI	HIHI	0.54
i1	F	HIHI	HIHI	HIHI	0.85	-	-	0.54	HIHI	HIHI	0.44
i2	i3	HIHI	-	HIHI	0.67	HIHI	HIHI	0.42	HIHI	HIHI	0.56
i2	i4	LOLO	LOLO	LOLO	0.82	LOLO	LOLO	0.65	-	-	0.47
i2	i5	HIHI	-	-	0.24	-	-	-0.02	-	HIHI	0.16
i2	i6	HIHI	HIHI	HIHI	0.66	HIHI	HIHI	0.45	HIHI	HIHI	0.65
i2	F	HIHI	HIHI	HIHI	0.67	HIHI	HIHI	0.46	HIHI	HIHI	0.58
i3	i4	LOLO	LOLO	LOLO	0.58	LOLO	LOLO	0.38	-	-	0.31
i3	i5	HIHI	HIHI	HIHI	0.35	HIHI	HIHI	0.23	-	-	0.33
i3	i6	EQ	EQ	EQ	1.00	EQ	EQ	0.76	-	-	0.59
i3	F	EQ	EQ	EQ	1,00	EQ	EQ	0.74	HIHI	HIHI	0.62
i4	i5	HIHI	-	-	0.20	-	-	0.22	-	-	-.07
i4	i6	HIHI	HIHI	HIHI	0.56	HIHI	HIHI	0.50	-	-	0.27
i4	F	HIHI	HIHI	HIHI	0.57	HIHI	HIHI	0.44	-	-	0.27
i5	i6	LOLO	LOLO	LOLO	0.35	-	LOLO	0.21	-	LOLO	0.26
i5	F	LOLO	LOLO	LOLO	0.36	-	LOLO	0.13	-	-	0.66
i6	F	EQ	EQ	EQ	1.00	LOLO	LOLO	0.69	LOLO	LOLO	0.66

Table 1. Relationships in Synthetic Data Set.

Input	Method	Sensitivity
Error-0	*Original-BR*	0.80
Error-0	*New-BR*	0.94
Error-2	*Original-BR*	0.67
Error-2	*New-BR*	0.84
Error-10	*Original-BR*	0.35
Error-10	*New-BR*	0.45

Table 2. Summary of Results on Synthetic Data Set.

out-performed *Original-BR* even as the error in the signal increased. However, as the error increased, the number of true positives detected by both methods dropped. Neither method picks up any false negatives and so the specificity is 100%.

On the other hand, correlation coefficients can be small even when a true implication exists. Furthermore, correlation is not at all informative about the type of implication (such as `HIHI` or `LOLO`). Consequently, the superset cannot be distinguished from the subset using correlation.

The results presented here indicate that Boolean implications are best suited for representing asymmetric relationships such as subset relationships. Furthermore, our proposed enhancements for Boolean implication extraction make the test more sensitive and help identify more true positives without picking up any false negatives.
