# Supplementary material for: Mining TCGA Data Using Boolean Implications
Source: PLoS One. 2014 Jul 23;9(7):e102119. doi: 10.1371/journal.pone.0102119 (PMC4108374; doi:10.1371/journal.pone.0102119)
Supplement: Text S3 — Mutual Exclusion Relations in the Genomic Alterations Sub-network. (PDF) [file pone.0102119.s021.pdf]

Mutual Exclusion Relations in the Genomic Alterations Subnetwork

HILO implications represent mutual exclusions and could indicate alternate hits on the same pathway. In our results (details are in Table S7), there was evidence for this possibility. Table 1 summarizes all the cases where the mutual exclusion relationships were between genes on pathways curated by the TCGA [1]. While many mutual exclusion relationships were between genes on the same pathway or closely coupled pathways (such as PI3K class 1 and RAS pathways or P53 and RB signalling pathways), there were mutual exclusion relationships between genes on different pathways (last two rows of Table 1). This pattern of mutual exclusion may highlight uncharacterized disease progression subgroups. Our graph also reveals mutual exclusion between IDH1 mutations and 7p11_2 amplifications and 10q23_31 deletions. These relations are probably subtype-specific occurrences since IDH1 mutations are associated with the G-CIMP group within the Proneural subtype and 7p11_2 (EGFR) amplifications and 10q23_31 (PTEN) deletions were associated with the Classical subtype [2]. As far as we know, this relationship has not been uncovered before. Some of these relationships may not have been identified earlier because of incomplete pathway information. This demonstrates the value of our unbiased method that does not depend on a priori information about pathways.

Alteration 1	Alteration 2	Possible Biological Significance
7p11_2-amp	11p15_5-del	EGFR and HRAS; RAS signalling
12q15-amp	TP53_mut	MDM2 and TP53; TP53 signalling
19q12-amp	13q14_2-del	CCNE1 and RB1; RB signalling
9p21_3-del	RB1_mut	CDKN2A/CDKN2B and RB1; RB signalling
ERBB2_M_mut	10q23_31-del	ERBB2 and PTEN; RAS and PI3K class 1 pathway
19q12-amp	TP53_mut	CCNE1 and TP53; RB signalling and P53 pathway
12q14_1-amp	PTEN_mut	CDK4 and PTEN; RB signalling and PI3K class1 signalling
13q14_2-del	EGFR_mut	RB1 and EGFR; RB signalling and RAS signalling

Table 1. Mapping the HILO implications in the GBM data set to known signalling pathways.

References

1. McLendon R, Friedman A, Bigner D, Van Meir E, Brat D, et al. (2008) Comprehensive genomic characterization defines human glioblastoma genes and core pathways. Nature 455: 1061-1068.

2. Verhaak R, Hoadley K, Purdom E, Wang V, Qi Y, et al. (2010) Integrated genomic analysis identifies clinically relevant subtypes of glioblastoma characterized by abnormalities in pdgfra, idh1, egfr, and nf1. Cancer Cell 17(1): 98-110.
